# Supplementary material for: Non-coding RNA LEVER sequestration of PRC2 can mediate long range gene regulation
Source: Commun Biol. 2022 Apr 11;5:343. doi: 10.1038/s42003-022-03250-x (PMC9001699; doi:10.1038/s42003-022-03250-x)
Supplement: Supplementary file 2 — Supplementary Information [file 42003_2022_3250_MOESM2_ESM.pdf]

## Supplementary Information

# Non-coding RNA *LEVER* sequestration of PRC2 can mediate long range gene regulation

Wei Wen Teo, Xinang Cao, Chan-Shuo Wu, Hong Kee Tan, Qiling Zhou, Chong Gao, Kim Vanuytsel, Sara S. Kumar, George J. Murphy, Henry Yang, Li Chai, and Daniel G. Tenen

- **Supplementary Figure 1-7 and legends**
- **Supplementary Table 1**

# Supplementary Figure 1

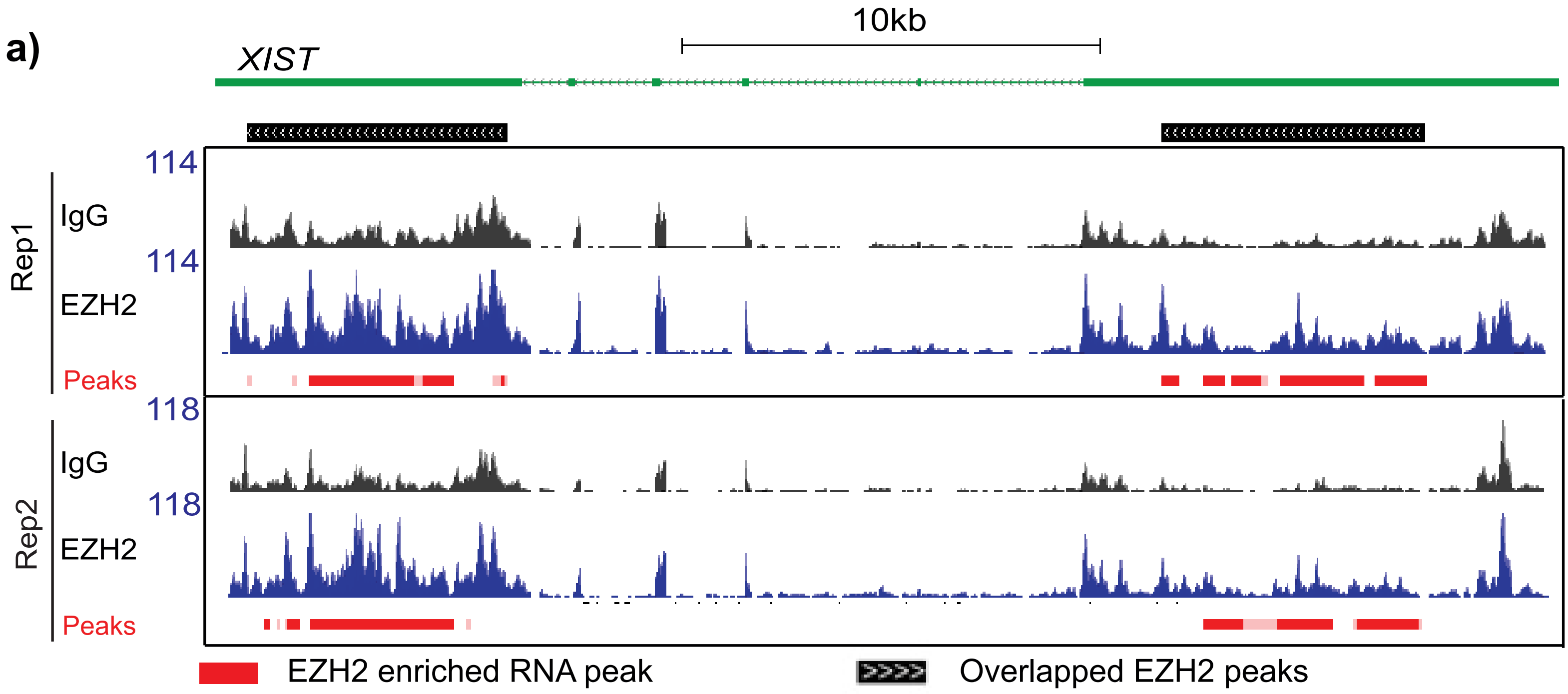


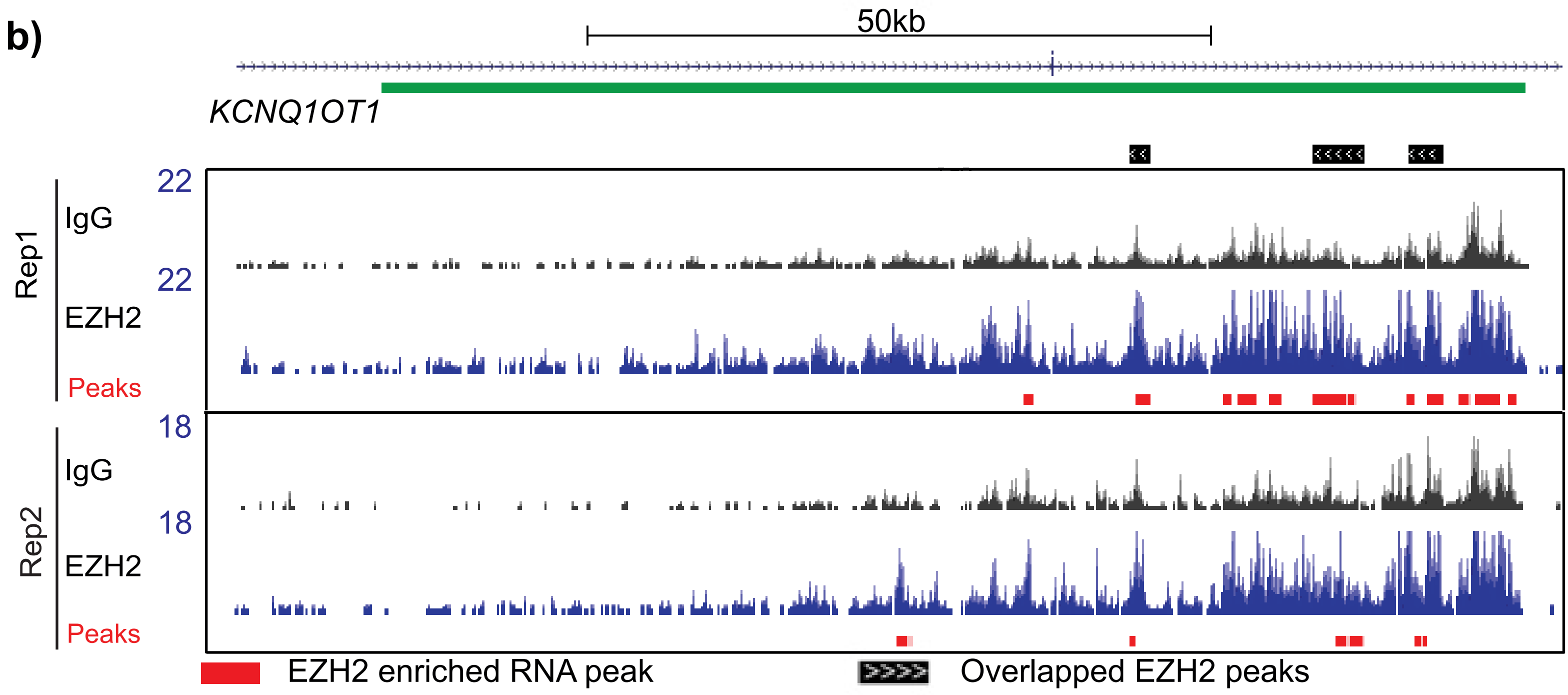

**Supplementary Figure 1. EZH2 RIP-seq enriched known EZH2-interacting lncRNAs.**

(a) Genome tracks of enriched EZH2 RIP peaks of XIST RNA with reference to Genecode v29 in K562 cells.
(b) Genome tracks of enriched EZH2 RIP peaks of KCNQ1OT1 RNA with reference to Genecode v29 in K562 cells.

# Supplementary Figure 2

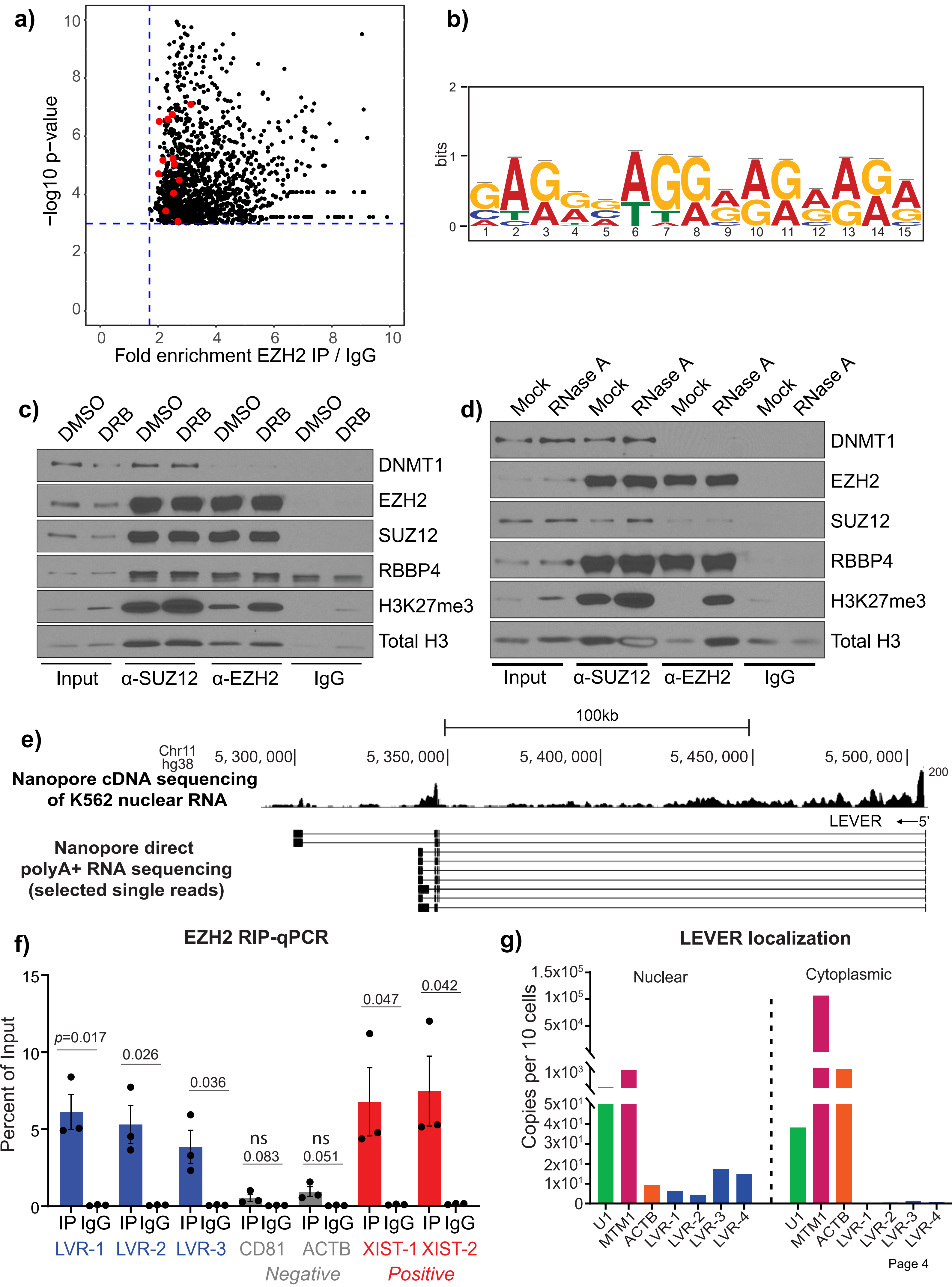

**Supplementary Figure 2. RNA-interacting properties of EZH2.**

(a) Fold enrichment – p-value plot of EZH2 RIP-seq identified peaks. One dot represents one individual peak. The 12 peaks annotated to *LEVER* transcript as described in Fig.1c are labelled as red dots.

(b) RNA motif identified from overlapped EZH2 peaks by MeMe motif discovery analysis. Two sets of flanking sequences were included in the analysis to eliminate background noise.

(c, d) EZH2 or SUZ12 co-immunoprecipitation (co-IP) in K562 cells treated with (c) 5,6-dichloro-1-b-D-ribofuranosylbenzimidazole (DRB) or (d) RNase A. Co-IP of PRC2 subunits (EZH2, SUZ12, RBBP4), PRC2-interacting protein (DNMT1), and histones (total histone H3 and histone H3 with H3K27me3 methylation) were measured by western blot. IgG isotype control antibody was used as co-IP negative control.

(e) Mapping of *LEVER* RNA to the human genome with Nanopore cDNA sequencing of K562 nuclear RNA or direct-RNA sequencing of K562 total polyA+ RNA.

(f) EZH2 RIP-qPCR on three *LEVER* fragments (LVR-1, LVR-2, and LVR-3, highlighted in Fig 1d), CD81 and ACTB (RIP-negative controls), and two XIST fragments (RIP-positive controls), quantified as percent of total input. Data from n=3 biological replicates are shown. Error bars represent SEM. Statistical difference was calculated using Welch's one-tailed t-test. ns, not significant.

(g) Digital droplet RT-PCR of *LEVER* fragments (LVR-1 through LVR-4), U1 and MTM1 (nuclear enriched), and ACTB (cytoplasmic enriched) RNAs in K562 nuclear or cytoplasmic RNA fractions.

# Supplementary Figure 3

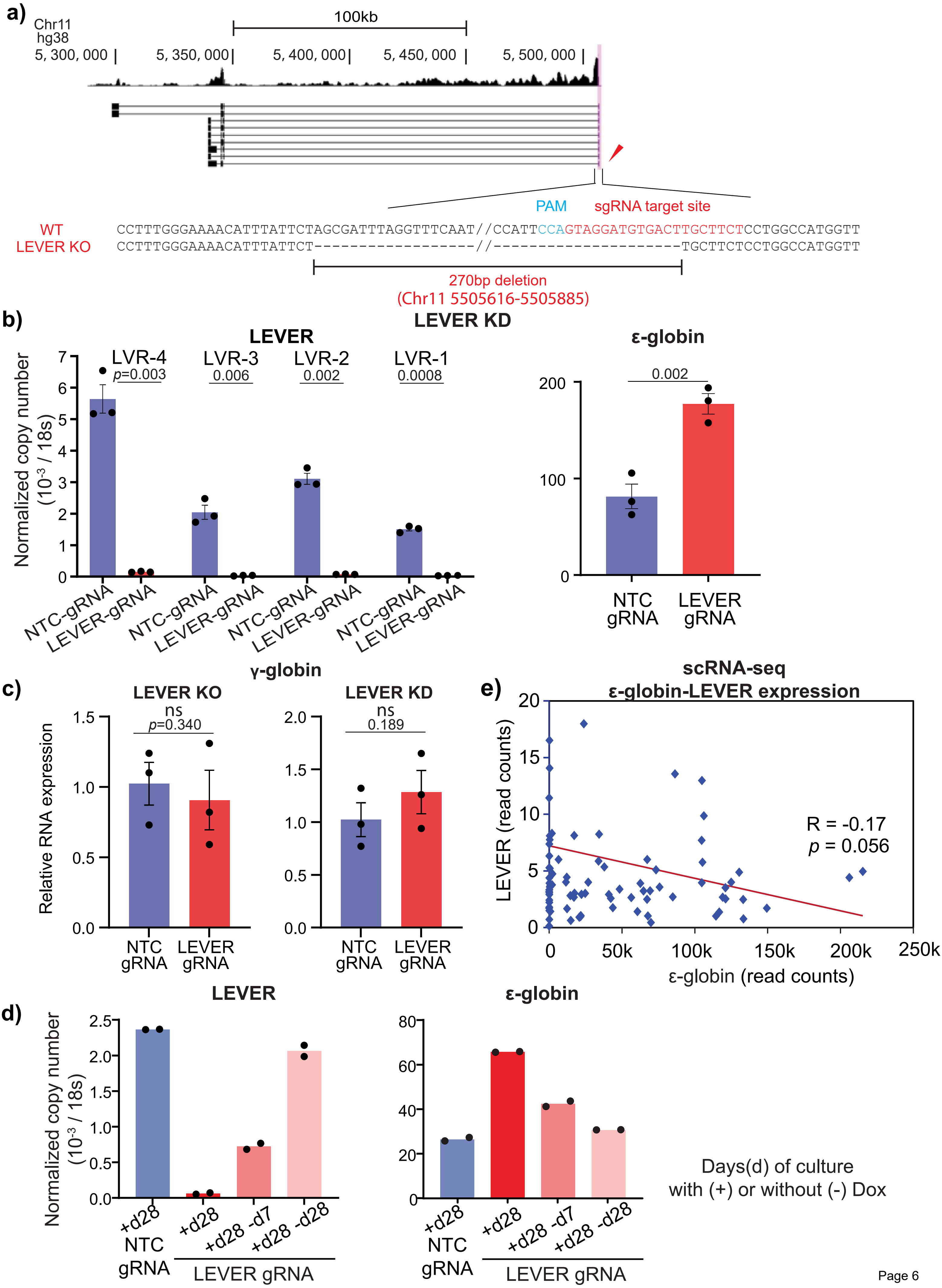

# Supplementary Figure 3 cont.

f)

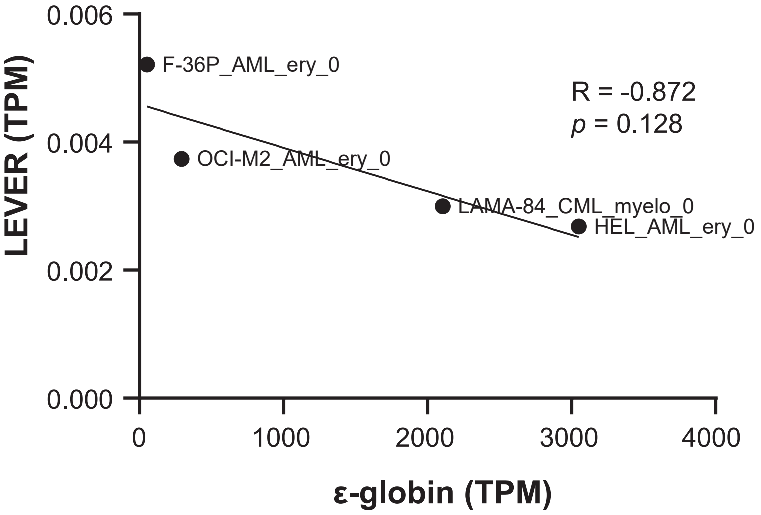


g)

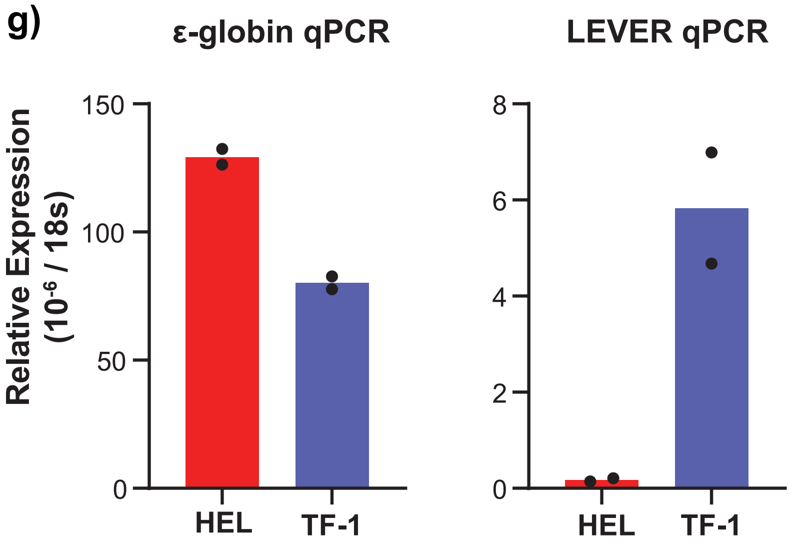

**Supplementary Figure 3. *LEVER* RNA negatively regulates *ε-globin*.**

(a) CRISPR-Cas9 generated *LEVER* KO cells with deletion spanning the *LEVER* promoter.

(b) *LEVER* and *ε-globin* expression after inducible *LEVER* knock-down (KD) in K562 cells measured by RT-qPCR. The cells were treated with dox for 28 days. Data from n=3 biological replicates are shown. Error bars represent SEM. Statistical difference was calculated using Welch's one-tailed t-test.

(c) *γ-globin* expression after inducible *LEVER* knock-out (KO) or *LEVER* knock-down (KD) in K562 cells measured by RT-qPCR. Data from n=3 biological replicates are shown. Error bars represent SEM. Statistical difference was calculated using Welch's one-tailed t-test. ns, not significant.

(d) *LEVER* and *ε-globin* expression in inducible *LEVER* KD K562 cells with (+) or without (-) doxycycline treatment for the indicated duration. *LEVER* expression is quantified at the LVR-4 region. Data from technical replicates of one biological sample is shown.

(e) Scatter plot showing normalized read counts of *ε-globin* (x-axis) and *LEVER* (y-axis) in the single-cell RNA-seq of human iPSC-derived erythroblasts. Each dot represents one single cell. The labelled correlation coefficient and *p*-value are generated by the Pearson correlative test.

(f) Scatter plot showing normalized read counts of *ε-globin* (x-axis) and *LEVER* (y-axis) in the RNA-seq of human *ε-globin* expressing lymphoma or leukemia cell lines. Data was obtained from Quentmeier et al [1]. The labelled correlation coefficient and *p*-value are generated by the Pearson correlative test.

**(g)** *LEVER* and *ε-globin* expression in HEL and TF-1 cells measured by RT-qPCR. Data from technical replicates of one biological sample is shown.

# Supplementary Figure 4

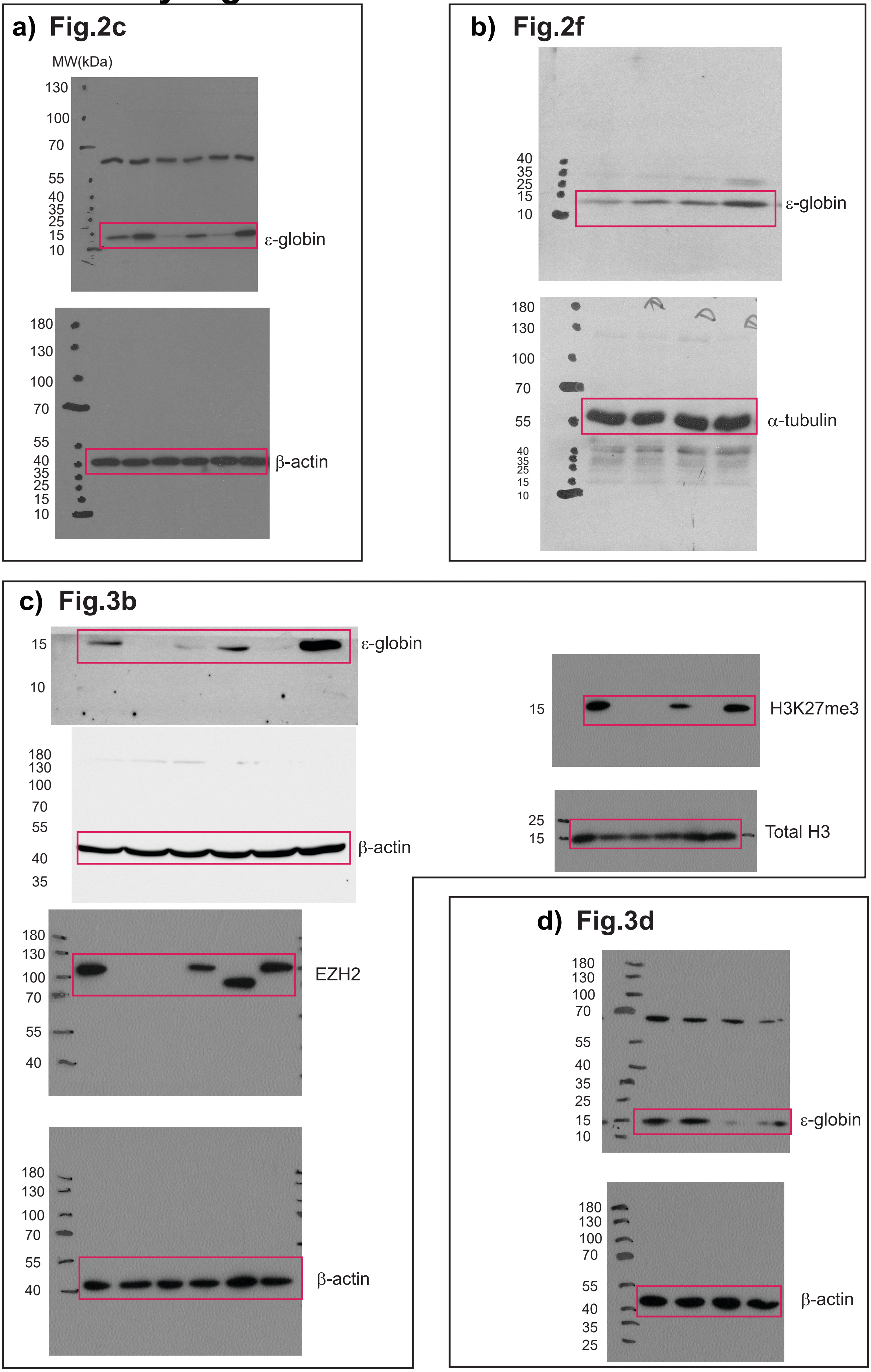

**Supplementary Figure 4. Uncropped western blots.**

Uncropped western blots of a) Fig.2c, b) Fig.2f, c) Fig.3b and d) Fig.3d.

# Supplementary Figure 5

**a)**

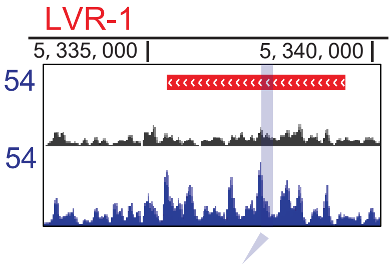


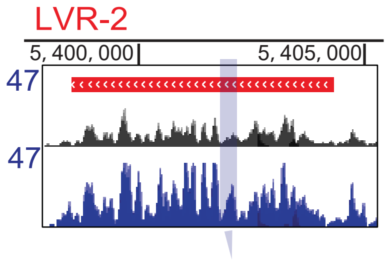


```
>chr11:5337598-5337847 (250nt)
CCTGTAATCTCAGCTACTCAGGAGGCTGAGGCAGGAGAATTGCTTGAACC
CAGGAGGCAGAGGTTGCAGTGAGCTGAGATCGCACCACTGCACTCTAGCA
TGGGTGAAAGAGTGAGACTTCATCTCAAAATAAAGAAAAATACAATTAAA
TACAGAGCTTCTAAACAGCAAAATAAACTATCATCAGAGCGAACAGGCAA
CCTACAGAATGGGAGAAAATTTCTGCAATCTACCCATCTGACAAAGGTCT
```

```
>chr11:5401788-5402156 (369nt)
GCCTGTAGTCCCAGCTACTTGAGAGGCTGAGGTGGGAAGATCACTTGGGC
CCAGAAGGCAAAGGTTGCAGTGAGCCAGGATTGCACGCCTACACTTATGC
CTGGGTAACAGCATGAGACAAAAGAAAAAGGAAGAGAAAAGAAAAGAAAG
AAAAAGAGAACAGCAAGCGGGAGGGAGGGAAGGAGGAAAGAAGGAAGGAA
AGAAGAGAGGAAAGAATAATAAAAGAAAAGGAAAGAAGAGAGAGAGAGAA
AGAAAAGGAAGGAAGGAAGAGAGAGAAAAGGAAGGAAGGAAAAAGAAAAA
GCAGAAGAAAAAGAGGGAAGGAAGGAATGATAGAAAAAGAAAGAAAAGAA
AGATTATGCATTTAAGGTG
```

**b)**

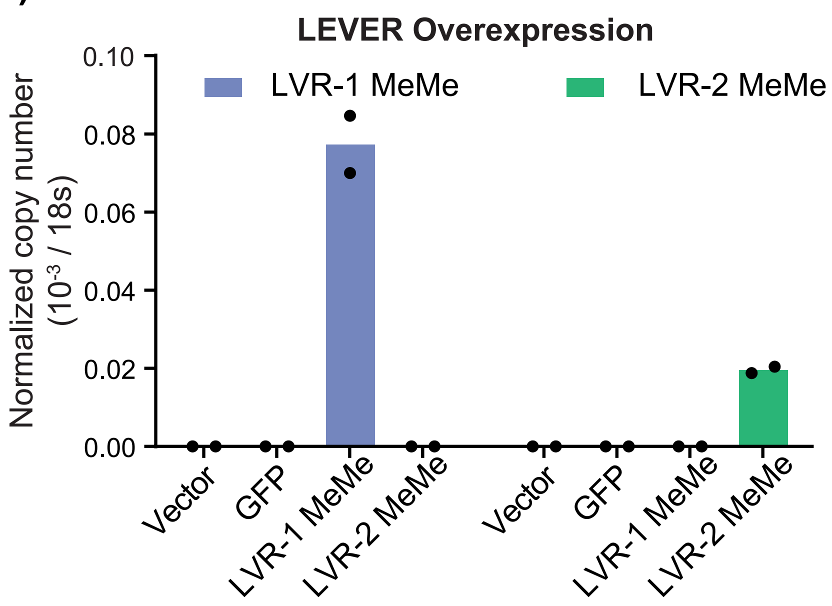

**Supplementary Figure 5. *LEVER*-MeMe overexpression.**

(a) Annotation and sequences of *LEVER*-MeMe fragments.
(b) RT-qPCR analysis of *LEVER*-MeMe fragments in *LEVER*-KO+Vector control, KO+GFP RNA control, KO+LVR-1 MeMe, and KO+LVR-2 MeMe cells. Data from technical replicates of one biological sample is shown.

# Supplementary Figure 6

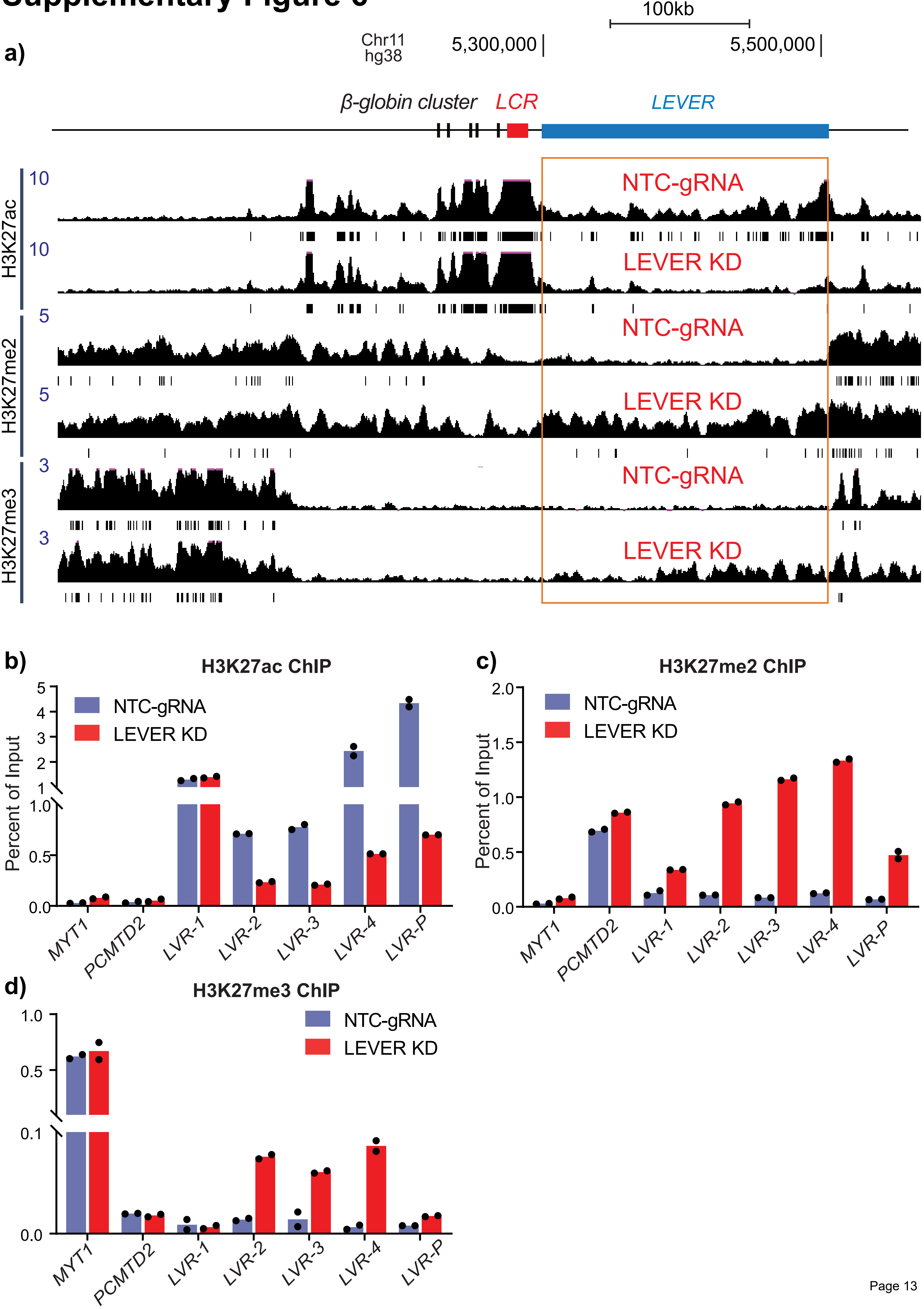

**Supplementary Figure 6. *LEVER* RNA sequesters EZH2 and blocks histone H3K27 methylation at the *LEVER* locus *in cis*.**

(a) Signal tracks and identified peaks of the ChIP-seq experiment in Fig.4c.
(b,c,d) ChIP-qPCR validation of the ChIP-seq experiment in Fig.4c. MYT1 serves as a ChIP positive control and PCMTD2 serves as a ChIP negative control. Data from technical replicates of one biological sample is shown.

# Supplementary Figure 7

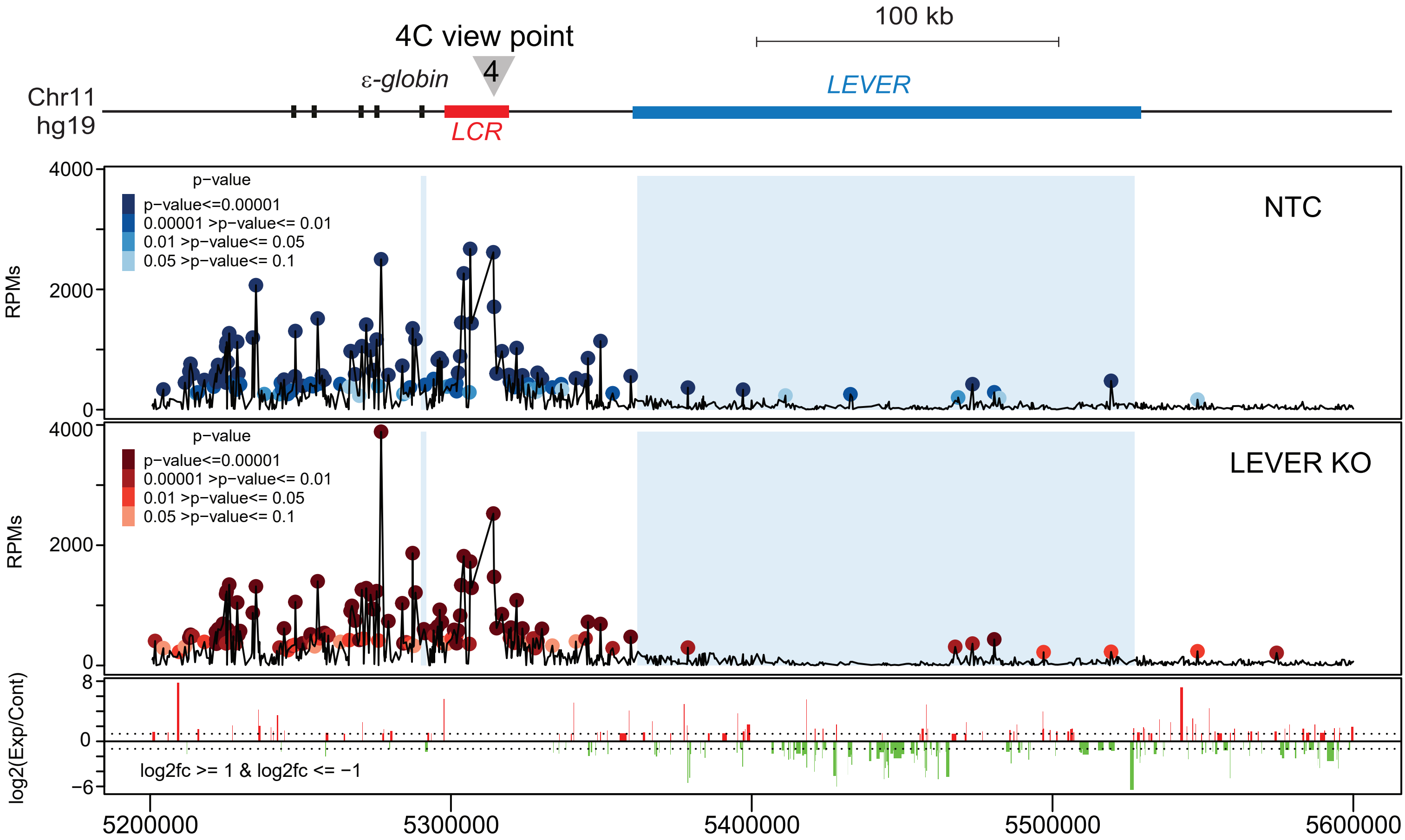

**Supplementary Figure 7. The *LEVER* locus regulates *ε-globin* through long-range chromatin interactions.**

A region within *LEVER* locus showed decreased interaction with LCR-HS4 upon *LEVER* knock out, as revealed by both reduced number of significant peaks (circles) and decreased signal levels (log2fc<-1). 4C-seq results of NTC and *LEVER* KO cells captured by viewpoint 4 (LCR-HS4) at the genomic region encompassing *β-globin* cluster, LCR and *LEVER* locus are shown. Data from two biological replicates were integrated. Normalized 4C signals (RPMs) at corresponding genomic locus were calculated within each restriction fragment (of DpnII). Reproducibly identified, statistically significant interacting regions are labelled by circles, with different colors representing different significance levels. The log2 fold changes (log2Exp/Cont) calculated between the 4C signal of *LEVER* KO (Exp) and NTC (Cont) cells were presented at the bottom. Only regions with log2fc >1 (red) and log2fc < -1 (green) were shown. This figure was shown in hg19 coordinates due to the requirements of the 4C analyzing package r3Cseq on this genome build.

## Supplementary Tables

### Supplementary Table 1. Antibodies used in this study

| Antibody (Clone/Cat#) | Supplier | RIP | ChIP | co-IP | Western |
|---|---|---|---|---|---|
| EZH2 (AC22, #39875) | Active Motif | 5μg | X | X | X |
| EZH2 (AC22, #3147) | Cell Signaling Technology | X | X | X | 1:1000 |
| EZH2 (D2C9, #5246) | Cell Signaling Technology | X | X | 2μg | X |
| EZH2 (#17-622) | Millipore | X | 1:50 | X | X |
| DNMT1(ab87656) | abcam | X | X | X | 1:1000 |
| SUZ12 (D39F6, #3737) | Cell Signaling Technology | X | X | 2μg | 1:1000 |
| RBBP4 (#4633) | Cell Signaling Technology | X | X | X | 1:1000 |
| H3K27Me3 (C36B11, #9733) | Cell Signaling Technology | X | 1:50 | X | 1:1000 |
| H3K27Me2 (D18C8, #9728) | Cell Signaling Technology | X | 1:50 | X | X |
| H3K27ac (ab4729) | abcam | X | 2μg | X | X |
| Histone H3 (1B1B2, #14269) | Cell Signaling Technology | X | X | X | 1:1000 |
| ε-globin (ab156041) | abcam | X | X | X | 1:500 |
| RNA Polymerase II (8WG16, ab817) | abcam | X | 4.5μg | X | X |
| β-actin (C4. sc-47778) | Santa Cruz | X | X | X | 1:2000 |
| α-tubulin | Santa Cruz | X | X | X | 1:1000 |
| goat anti-rabbit IgG-HRP (sc-2004) | Santa Cruz | X | X | X | 1:2000 |
| goat anti-mouse IgG-HRP (sc-2005) | Santa Cruz | X | X | X | 1:2000 |
| normal Rabbit IgG (sc-2027X) | Santa Cruz | Assay Dependent | | | |
| Rabbit IgG monoclonal (ab172730) | Santa Cruz | | | | |
| Mouse IgG monoclonal (#5415) | Cell Signaling Technology | | | | |

## References

1 Quentmeier, H. *et al.* The LL-100 panel: 100 cell lines for blood cancer studies. *Sci Rep* **9**, 8218, doi:10.1038/s41598-019-44491-x (2019).
