# Supplementary material for: Non-coding RNA LEVER sequestration of PRC2 can mediate long range gene regulation
Source: Commun Biol. 2022 Apr 11;5:343. doi: 10.1038/s42003-022-03250-x (PMC9001699; doi:10.1038/s42003-022-03250-x)
Supplement: Supplementary file 3 — Description of Additional Supplementary Files [file 42003_2022_3250_MOESM3_ESM.pdf]

Description of Additional Supplementary Files

File name: Supplementary data 1
Description: Plasmids, primers, and sgRNA sequences used in this study

File name: Supplementary data 2
Description: Source data of main figures.
