# Supplementary material for: Non-coding RNA LEVER sequestration of PRC2 can mediate long range gene regulation
Source: Commun Biol. 2022 Apr 11;5:343. doi: 10.1038/s42003-022-03250-x (PMC9001699; doi:10.1038/s42003-022-03250-x)
Supplement: Supplementary file 6 — Reporting Summary [file 42003_2022_3250_MOESM6_ESM.pdf]

nature research

Corresponding author(s): Li Chai, Daniel Tenen

Last updated by author(s): Feb 16 2022

Reporting Summary

Nature Research wishes to improve the reproducibility of the work that we publish. This form provides structure for consistency and transparency in reporting. For further information on Nature Research policies, see our Editorial Policies and the Editorial Policy Checklist.

Statistics

For all statistical analyses, confirm that the following items are present in the figure legend, table legend, main text, or Methods section.

n/a	Confirmed	
☐	☒	The exact sample size (*n*) for each experimental group/condition, given as a discrete number and unit of measurement
☐	☒	A statement on whether measurements were taken from distinct samples or whether the same sample was measured repeatedly
☐	☒	The statistical test(s) used AND whether they are one- or two-sided<br>*Only common tests should be described solely by name; describe more complex techniques in the Methods section.*
☒	☐	A description of all covariates tested
☒	☐	A description of any assumptions or corrections, such as tests of normality and adjustment for multiple comparisons
☒	☐	A full description of the statistical parameters including central tendency (e.g. means) or other basic estimates (e.g. regression coefficient) AND variation (e.g. standard deviation) or associated estimates of uncertainty (e.g. confidence intervals)
☒	☐	For null hypothesis testing, the test statistic (e.g. *F*, *t*, *r*) with confidence intervals, effect sizes, degrees of freedom and *P* value noted<br>*Give P values as exact values whenever suitable.*
☒	☐	For Bayesian analysis, information on the choice of priors and Markov chain Monte Carlo settings
☒	☐	For hierarchical and complex designs, identification of the appropriate level for tests and full reporting of outcomes
☒	☐	Estimates of effect sizes (e.g. Cohen's *d*, Pearson's *r*), indicating how they were calculated

Our web collection on statistics for biologists contains articles on many of the points above.

Software and code

Policy information about availability of computer code

Data collection	Illumina bcl2fastq2 Conversion Software (v2.20) was used for Next Generation Sequencing library demultiplexing. MinKNOW was used to collect Nanopore Sequencing raw reads. Albacore (v2.3.3) was used to convert Nanopore raw reads to FASTQ format.
Data analysis	bowtie2,SAMtools,BEDTools,bedGraphToBigWig,MACS2,TrimGalore,STAR,cuffdiff,featureCounts,MEME, Minimap2, bamToBed,LAST, maf-convert r3CSeq (v1.30.0), CrossMap, deepTools, GraphPad Prism.

For manuscripts utilizing custom algorithms or software that are central to the research but not yet described in published literature, software must be made available to editors and reviewers. We strongly encourage code deposition in a community repository (e.g. GitHub). See the Nature Research guidelines for submitting code & software for further information.

Data

Policy information about availability of data

All manuscripts must include a data availability statement. This statement should provide the following information, where applicable:

- Accession codes, unique identifiers, or web links for publicly available datasets
- A list of figures that have associated raw data
- A description of any restrictions on data availability

The ChIP-seq, RNA-seq, 4C-seq and Nanopore sequencing data in this study have been deposited in GEO under accession number GSE141083 [https://www.ncbi.nlm.nih.gov/geo/query/acc.cgi?acc=GSE141083].

Field-specific reporting

Please select the one below that is the best fit for your research. If you are not sure, read the appropriate sections before making your selection.

☒ Life sciences ☐ Behavioural & social sciences ☐ Ecological, evolutionary & environmental sciences

For a reference copy of the document with all sections, see nature.com/documents/nr-reporting-summary-flat.pdf

Life sciences study design

All studies must disclose on these points even when the disclosure is negative.

Sample size	No statistical methods were used to predetermine sample size.
Data exclusions	No data were excluded from the analyses.
Replication	Three biological replicates were tested for most experiments unless otherwise labeled in the figure legends.
Randomization	No method of randomization was used as this is not relevant to the assays used in this study.
Blinding	No blinding was applied as all data are quantitative and not easily subjected to investigator bias.

Reporting for specific materials, systems and methods

We require information from authors about some types of materials, experimental systems and methods used in many studies. Here, indicate whether each material, system or method listed is relevant to your study. If you are not sure if a list item applies to your research, read the appropriate section before selecting a response.

Materials & experimental systems

n/a	Involved in the study	
☐	☒	Antibodies
☐	☒	Eukaryotic cell lines
☒	☐	Palaeontology and archaeology
☒	☐	Animals and other organisms
☒	☐	Human research participants
☒	☐	Clinical data
☒	☐	Dual use research of concern

Methods

n/a	Involved in the study	
☐	☒	ChIP-seq
☒	☐	Flow cytometry
☒	☐	MRI-based neuroimaging

Antibodies

Antibodies used	All antibodies used in this study were listed with RRID in supplementary table 1.
Validation	https://www.thermofisher.com/antibody/product/EZH2-Antibody-clone-AC22-Monoclonal/39875<br>https://www.cellsignal.com/products/primary-antibodies/ezh2-ac22-mouse-mab/3147<br>https://www.cellsignal.com/products/primary-antibodies/ezh2-d2c9-xp-rabbit-mab/5246<br>https://www.abcam.com/dnmt1-antibody-ab87654.html<br>https://www.cellsignal.com/products/primary-antibodies/suz12-d39f6-xp-rabbit-mab/3737<br>https://www.cellsignal.com/products/primary-antibodies/rbap46-rbap48-antibody/4633<br>https://www.cellsignal.com/products/primary-antibodies/tri-methyl-histone-h3-lys27-c36b11-rabbit-mab/9733<br>https://www.cellsignal.com/products/primary-antibodies/di-methyl-histone-h3-lys27-d18c8-xp-rabbit-mab/9728<br>https://www.abcam.com/histone-h3-acetyl-k27-antibody-chip-grade-ab4729.html<br>https://www.cellsignal.com/products/primary-antibodies/histone-h3-1b1b2-mouse-mab/14269<br>https://www.abcam.com/rna-polymerase-ii-ctd-repeat-ysptsps-antibody-8wg16-chip-grade-ab817.html

Eukaryotic cell lines

Policy information about cell lines

Cell line source(s)	K562 cell line was purchased from ATCC
Authentication	Cell lines were not authenticated.

Mycoplasma contamination	Cell lines tested negative for mycoplasma.
Commonly misidentified lines (See ICLAC register)	No commonly misidentified cell lines were used.

ChIP-seq

Data deposition

☒ Confirm that both raw and final processed data have been deposited in a public database such as GEO.

☐ Confirm that you have deposited or provided access to graph files (e.g. BED files) for the called peaks.

Data access links *May remain private before publication.*	https://www.ncbi.nlm.nih.gov/geo/query/acc.cgi?acc=GSE141083<br>token: mlchaysqffuhjen
Files in database submission	A full list of file can be found under the accession numbers provided above.
Genome browser session (e.g. UCSC)	https://genome.ucsc.edu/s/piggy_cher/hg38_LEVER_submission

Methodology

Replicates	ChIP-seq is performed from one biological sample.
Sequencing depth	ntgRNA-Input 51327391 40010826 36 paired-end<br>ntgRNA-H3K27Ac 31526063 25791756 36 paired-end<br>ntgRNA-H3K27Me2 44057810 33591417 36 paired-end<br>ntgRNA-H3K27Me3 34047315 26802964 36 paired-end<br>LEVER_KD-Input 65046035 51114679 36 paired-end<br>LEVER_KD-H3K27Ac 26799955 22060877 36 paired-end<br>LEVER_KD-H3K27Me2 32662615 25168367 36 paired-end<br>LEVER_KD-H3K27Me3 35877350 28336419 36 paired-end
Antibodies	H3K27Me3 (Cell Signaling Technology,C36B11, #9733)<br>H3K27Me2 (Cell Signaling Technology, D18C8, #9728)<br>H3K27ac (abcam, ab4729)
Peak calling parameters	Samples were aligned to the human genome (build hg38, GRCh38) with bowtie2 with default settings. Then, non-duplicate mapped reads with MAPQ>20 were retained using Samtools. Peaks were called by MACS2. Sharp peaks with q-value cutoff 0.01 or 0.05 were generated separately with the following argument: "--keep-dup all"; broad peaks were generated similarly with default broad-region cut-off (0.1).
Data quality	Number of peaks with fold enrichment > 5 and FDR<0.05 (broad peaks):<br>ntgRNA-H3K27Ac 12333<br>ntgRNA-H3K27Me2 9<br>ntgRNA-H3K27Me3 511<br><br>LEVER_KD-H3K27Ac 12855<br>LEVER_KD-H3K27Me2 7<br>LEVER_KD-H3K27 286
Software	TrimGalore, bowtie2, SAMtools, BEDTools, bedGraphToBigWig, MACS2
